# Amylin: A Multi‐Functional Pancreatic Hormone—A Review

**DOI:** 10.1111/dom.71146

**Published:** 2026-08-05

**Authors:** Christelle Le Foll, Thomas A. Lutz

**Affiliations:** ^1^ Institute of Veterinary Physiology, Vetsuisse Faculty University of Zurich Zurich Switzerland; ^2^ One Health Institute University of Zurich Zurich Switzerland

**Keywords:** anti‐obesity drug, appetite control, GLP‐1, Type 2 diabetes

## Abstract

Amylin is a pancreatic beta‐cell hormone that sits at a systems hub connecting key aspects of energy metabolism. Amylin physiologically controls satiation, slows gastric emptying and limits postprandial glucagon release, while its actions also link homeostatic eating controls with reward driven behaviours via defined central nervous system pathways. Further, at least under certain conditions, aggregating amylin exerts a strong pathophysiological impact on the destruction of pancreatic beta‐cells in Type 2 diabetes mellitus (T2D), providing a plausible explanation for why a T2D‐like disease entity is only observed in a few mammalian species. This review will provide a brief summary of selected aspects of our current understanding of the biology of amylin and outlines how this knowledge has recently been translated into effective pharmacotherapy for obesity and diabetes, including long‐acting analogs combination approaches with other peptide hormones.

## Plain Language Summary

- Amylin, also called islet amyloid polypeptide, is a pancreatic beta‐cell hormone that has been discovered approx. 40 years ago.
- The original discovery was made on the basis of the pathophysiological finding of amyloid deposits in the pancreas of individuals suffering from Type 2 diabetes mellitus.
- Shortly after, however, it also became clear that this aggregation of amylin into amyloid only occurs under certain conditions, and that amylin in fact has many beneficial hormonal effects to control metabolism.
- The best studied effects in this respect are the control of eating and of glucose metabolism.
- Amylin's effects seem to be mediated by direct action in the brain.
- These physiological metabolic effects build the biological basis for the development of highly efficacious drugs to treat obesity and Type 2 diabetes mellitus.
- Take home messages: (1) amylin is co‐secreted with insulin; (2) under some conditions, human amylin can aggregate and damage beta‐cells; (3) circulating amylin is a hormone that controls eating and glucose metabolism; (4) amylin analogs are most promising anti‐obesity drugs currently in clinical testing.

## 1 Introduction: From an Islet Co‐Hormone to a Systems Node in Physiology and Pathophysiology

This review will provide a brief summary of selected aspects of our current understanding of the biology of amylin. The authors are aware of the fact that several seminal reviews on amylin have been published recently by authors who themselves have published a lot on amylin biology [1, 2, 3, 4]. Here, in addition to the important topic of amylin receptor biology, we focus also on developmental aspects of amylin and a more integrative view on its role in controlling metabolism.

The pancreatic beta‐cell hormone amylin sits at a central systems hub controlling various key aspects of metabolism, including eating control, glycaemic control, energy balance and connects homeostatic with hedonic feeding. Interestingly, in the case of amylin, glycaemic control shows an important dichotomy of effects because beneficial effects of monomeric, circulating amylin contrast with the ability of amylin to aggregate under some conditions, leading to beta‐cell destruction that eventually will lead to Type 2 diabetes mellitus (T2D) [2, 5]. Both opposing actions of this biology have led to the development of highly interesting therapeutic options, on one side (long‐acting) amylin analogs for the treatment of obesity and T2D with some analogs having completed Phase III clinical trials [6] and on the other side ways to interfere with amylin aggregation to preserve functional beta‐cell mass in T2D [7].

**FIGURE 1:**
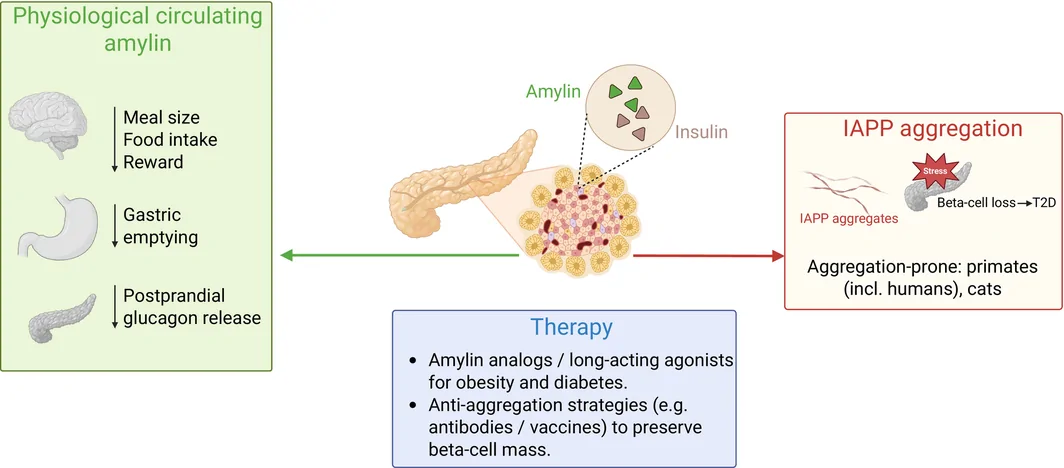
Amylin as a systems hub in physiology and pathophysiology. Amylin is co‐synthesised and co‐secreted with insulin from pancreatic beta‐cells and circulating monomeric amylin acts as a systems‐level regulator of nutrient influx by inducing satiation, slowing gastric emptying, limiting postprandial glucagon secretion and modulating reward‐related feeding behaviour via defined brainstem and hypothalamic pathways. On the other hand, under aggregation‐prone conditions in certain species, amylin (for that reason also called islet amyloid polypeptide, IAPP) forms toxic aggregates in the islet, contributing to beta‐cell dysfunction and loss, namely in primates and cats, thereby promoting T2D. This duality has inspired both the development of long‐acting amylin analogs for metabolic disease and anti‐aggregation strategies aiming to preserve beta‐cell mass.

Amylin and insulin can be considered sister hormones, being co‐expressed and co‐secreted from the same secretory granules of pancreatic beta‐cells [8, 9, 10]. Glucose is the primary regulator of amylin synthesis and secretion. Amylin was originally named diabetes‐associated peptide and later islet amyloid polypeptide (IAPP) [11, 12]. Today, the term ‘amylin’ is commonly used for the circulating mature (monomeric) form of the 37‐amino‐acid peptide, while ‘IAPP’ is often used in the context of amylin aggregation, hence the fibrillating forms of the peptide [13] (Figure 1).

IAPP was identified in the mid 1980's as the main constituent of the typical deposits in the islet of Langerhans in people with T2D and felines with diabetes mellitus. It soon became clear that IAPP aggregation occurs only in a few mammalian species; the propensity of amylin to aggregate depends on slight differences in the amino acid sequence of the molecule [14]. This happens namely in primates (including humans) and in cats. This propensity to aggregate coincides with the occurrence of T2D‐like disease in the same species [15, 16, 17]. Hence, IAPP aggregation is a pathological feature but also a driving factor of T2D. Recently, therapeutic strategies have emerged, including antibody‐based vaccines that interfere with IAPP aggregation. These approaches show promise for the development of a new class of T2D therapies aimed at slowing down aggregation or potentially reversing the load of aggregated IAPP in order to restore functional beta‐cell mass [7, 18, 19]. The reader is referred to some seminal papers which cover this topic in much more detail [14, 20, 21, 22].

For the rest of this review, we will now mainly discuss the metabolic features of circulating amylin as a hormone that complements the metabolic effects of its sister hormone insulin. Generally speaking, amylin controls nutrient influx into the systemic ‘nutrient pool’ while insulin controls the utilisation of nutrients out of this pool [2, 13, 23]. Given their co‐synthesis and co‐secretion, it is not surprising that amylin's circulation patterns reflect those of insulin [24, 25]. Amylin induces satiation but also slows gastric emptying and reduces postprandial glucagon release; the latter two effects help to contribute to the attenuation of postprandial glucose excursions. These three actions of amylin were identified early after the discovery of amylin as a circulating hormone and they are still considered the best established physiological effects of amylin [26, 27, 28, 29, 30, 31, 32, 33, 34]. At the same time, these core effects are fundamental for the development of amylin analogs for the treatment of metabolic diseases, with the first (and so far only approved) amylin analog, pramlintide, having been licenced more than 20 years ago [2, 3, 6].

## 2 Amylin in Brain Development

**FIGURE 2:**
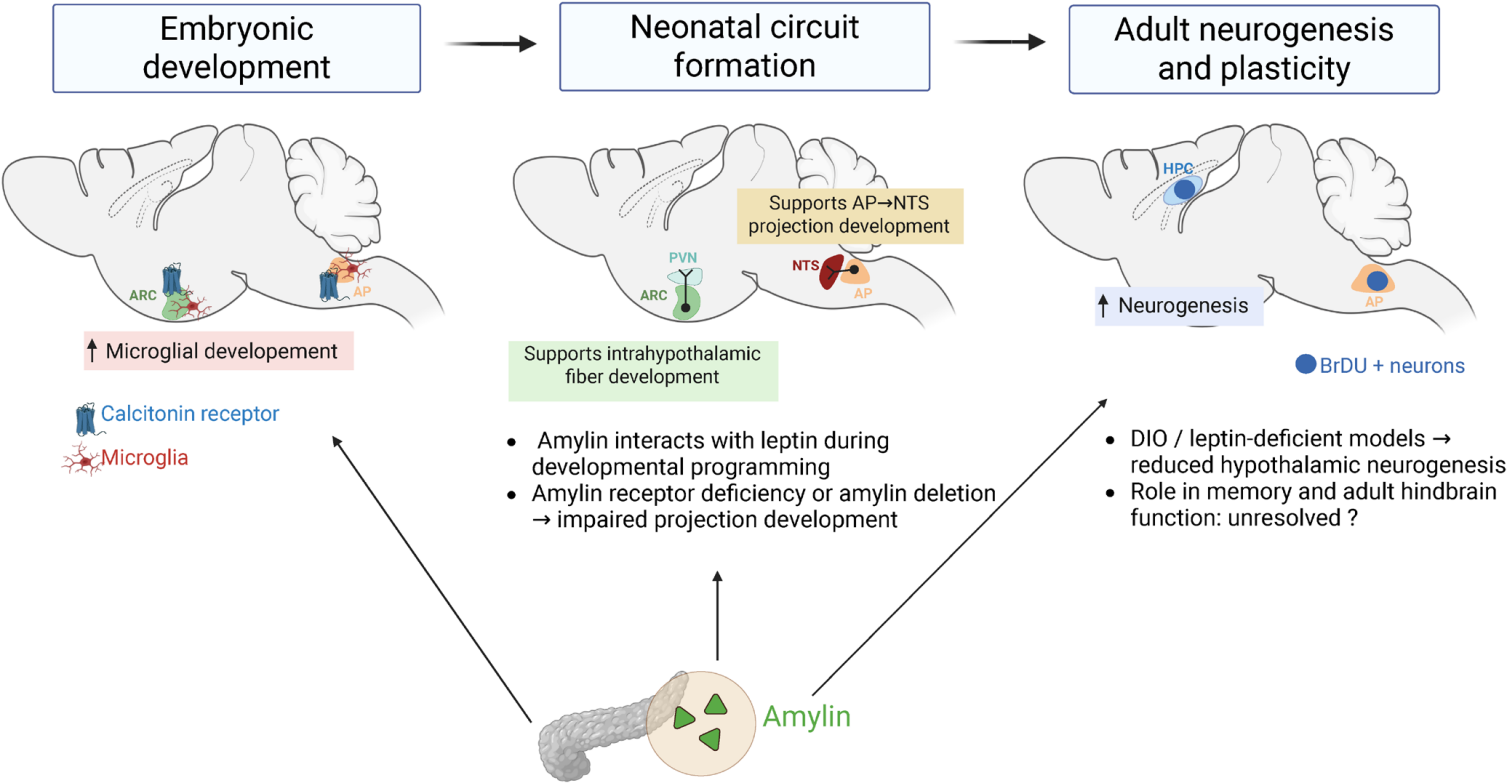
Roles of amylin in brain development and adult neurogenesis. During foetal and early postnatal life, amylin produced by the developing pancreas together with early expression of the CTR in the hypothalamus and caudal hindbrain contributes to microglial development and the formation of hypothalamic and AP–NTS projections that underlie later energy homeostasis. In adulthood, amylin, together with other metabolic hormones such as leptin, enhances neurogenesis in specific brain regions including the AP and possibly the hippocampus, with these processes being sensitive to the metabolic state, although the precise functional implications of amylin‐induced neurogenesis in the adult hindbrain remain to be defined.

As discussed in the following paragraphs, amylin's metabolic effects seem to be largely brain mediated rather than by direct action on peripheral tissues. In this context, it is interesting that amylin may in fact also impact brain development during different phases of development, thereby potentially shaping its own target tissue circuitry in adult life (Figure 2).

During embryogenesis, neurons in the mediobasal hypothalamus are born between embryonic day (E) 11 and E16 [35, 36]. Less is known about the exact time frames in the caudal hindbrain, including the area postrema (AP), but noradrenergic neurons which are targeted by amylin [37] most likely develop around the same time [38]. The expression of the calcitonin receptor (CTR) which constitutes the core receptor of the amylin receptor (see below) is also expressed early during brain development in various brain regions, including the hypothalamus and the caudal hindbrain [39]. Amylin itself is also expressed early in the foetal pancreas and peak secretions have been observed around day E17 [40, 41]. Hence, a functional amylin system is present early on in brain development. Interestingly, during this early developmental phase, the effect of amylin seems to affect more the development of microglia than of neurons in the hypothalamus and the AP [42]. This observation is particularly relevant given the role of microglia in mediating the sensitising effect of amylin on the catabolic effect of leptin, which will be discussed later in this review [43, 44, 45].

Amylin may also influence the neuronal pathway development during the neonatal period. In rodents, this pathway development takes place during the last week of gestation and during the first 2 weeks of life for a rodent [46, 47], possibly corresponding to the first 6 months of human life [48]. Leptin is very well known to control this developmental window; for example, leptin‐deficient ob/ob mice present a strong disruption in the development of intra‐hypothalamic projections which could be rescued by leptin replacement early in life [49]. Similar phenomena have also been seen in selectively bred diet‐induced obese (DIO) rats, likely reflecting early leptin resistance [43].

Amylin seems to be equally important for the normal formation of hypothalamic networks that are involved in the control of energy homeostasis. Amylin receptor deficient mice exhibit a decrease in the development of intrahypothalamic fibre projections indicating that amylin also acts as a neurotrophic factor during the first 2 weeks of life in the hypothalamus [45, 50, 51]. Beyond the hypothalamus, amylin also seems to be important for the normal formation of neuronal connections in the caudal hindbrain, in particular from the AP to the nucleus of the solitary tract (NTS) [52]. Neurite outgrowth from the AP to the NTS is decreased in amylin KO mice during the early postnatal period but, interestingly, this deficit persists into adulthood.

Neurogenesis is not limited to the embryonic period but proceeds also in adulthood in specific brain regions, in particular the hippocampus [53, 54]. Interestingly, metabolic hormones like amylin, leptin and insulin seem to enhance adult neurogenesis across different brain areas [47, 55]. This process also seems to be sensitive to the metabolic state: DIO mice displayed lower numbers of newly born cells compared to chow diet‐fed mice in the hypothalamic arcuate nucleus (ARC) [56]. In DIO mice, the differentiation of stem cells into proliferative progenitor‐like cells seemed to be inhibited, paralleled by increased apoptosis. Neurogenesis was even more reduced in adult ob/ob mice than in DIO mice, confirming the crucial role of leptin in neurogenic processes also in adulthood [56]. In the hippocampus, chronic leptin administration increased cell proliferation in adult mice without altering the differentiation and survival rate of these newly proliferated cells. Such mechanisms may perhaps be involved in the improvement of spatial memory and learning by leptin [57, 58]. Amylin also increases memory function in rats and mice [59] but it is unclear if this process is mediated by changes in neurogenesis in the hippocampus [60].

Amylin also seems to influence brain development processes in the adult caudal hindbrain, a region central to amylin's metabolic effects [1, 61]. Amylin upregulates genes such as NeuroD1 that are involved in cell proliferation and neurogenesis [55] and it increased neurogenesis, based on BrdU staining, in the AP of adult rats [55]. The precise functional implications of this effect still need to be determined.

## 3 Amylin Receptor Diversity, Bias and Plasticity

Amylin binds to three amylin receptors (AMYRs), which belong to the seven‐transmembrane domain G‐protein coupled receptors, specifically the CTR family [62, 63]. AMYRs are heterodimeric complexes composed of the CTR and a receptor activity‐modifying protein (RAMP), specifically RAMP1, RAMP2 or RAMP3 [62, 63]. The resulting three amylin receptors—AMY1R, AMY2R and AMY3R—may be associated with different biological functions although evidence for subtype‐specific effects is still scarce [64, 65, 66]. Current data suggest that AMY1R and AMY3R seem to be primarily associated with the inhibition of food intake and glucose regulation in contrast to AMY2R, whose role remains largely unexplored. Amylin also binds the CTR in the absence of a RAMP component, albeit at lower affinity. Both amylin and the amylin analog pramlintide transiently lower calcium levels in rodents [67] likely through activation of the CTR in osteoblasts and of the CTR or AMYR in osteoclasts. The physiological relevance of the CTR alone, that is, CTR‐mediated—and not AMYR‐mediated—signalling for the metabolic effects (i.e., reduction in eating, increase in energy expenditure and control of body weight) remains unclear [62, 63].

**FIGURE 3:**
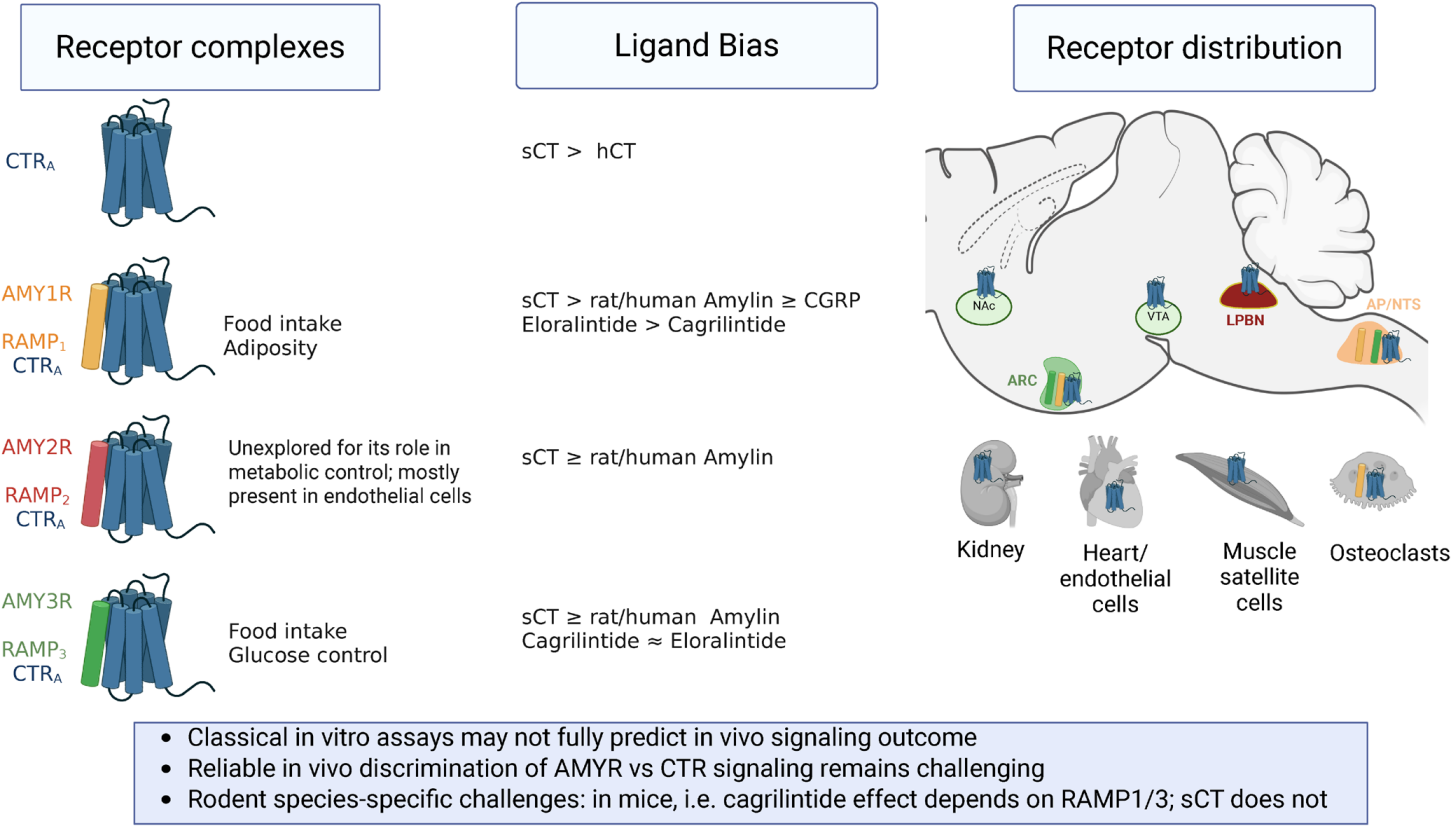
Amylin receptor diversity, ligand bias and tissue distribution. Amylin receptors are formed by heterodimeric complexes of the CTR with RAMP1–3, giving rise to AMY1R, AMY2R and AMY3R; CTR (the best characterised being the insert‐negative form of the CTR, i.e., CTR_a_) alone can also bind amylin with lower affinity. Structural and pharmacological data indicate that different ligands (amylin, pramlintide, cagrilintide versus calcitonin and salmon calcitonin) preferentially stabilise RAMP‐associated or CTR‐biassed conformations, while anatomical studies highlight strong evidence for functional receptor expression in the AP, NTS and hypothalamus and more limited, less well‐characterised co‐expression in peripheral tissues such as kidney, heart, skeletal muscle satellite cells and bone.

In recent years, several laboratories extensively characterised the complex interaction of amylin, calcitonin and their analogs with CTR and AMYR receptor complexes but the structural complexity of the AMYR and the lack of specific antibodies for the AMYRs challenge the in vivo characterisation of ligand receptor interaction and receptor distribution [62, 68]. Studies using cryogenic electron microscopy have shown that different AMYR and CTR agonists interact differently with AMYRs and the CTR [62, 63, 68, 69, 70]. For example, amylin and its analogs, such as cagrilintide and pramlintide, preferentially stabilise RAMP‐associated AMYR conformations, while calcitonin and salmon calcitonin (sCT) kept the receptor complex in a CTR‐biassed conformation. This finding was surprising given that the in vitro characteristics of cagrilintide and sCT, using a large array of classical cell based assays, looked rather similar [63]; however, the in vivo effects of the two peptides, at least in mice, differed markedly. In mice, the effect of cagrilintide, but not sCT, depended on the presence of RAMP1 and RAMP3 [71]. Hence, the ligand structure may also determine whether receptor activation yields an amylin‐dominant or calcitonin‐dominant signalling profile, potentially leading to different physiological outcomes that are not necessarily predicted by classical in vitro receptor binding or potency assays. Further, reliable differentiation of AMYR versus CTR activation may only be possible by testing ligands with preferred selectivity for either of these receptors. For these and other reasons, AMYR pharmacology remains a challenge (Figure 3). It also needs to be stressed that the CTR itself comes in various splice variants; most studies have been performed with the insert‐negative splice variant, the CTR_a_ [72], increasing receptor complexity even more.

### 3.1 Distribution of CTR/RAMPs

Most of our current understanding of AMYR distribution is derived from preclinical studies in rats and mice, for example, utilising approaches such as radioligand binding with labelled amylin and from gene expression analyses of CALCR, the gene encoding the CTR, and RAMPs within the same tissues [73, 74, 75]. Both the CTR and RAMPs, the latter based on mRNA data only, are widely expressed throughout the brain [76, 77], but obviously, co‐expression of both the CALCR and a RAMP within the same cell is necessary to generate a functional AMYR complex. This has in fact only been studied in very few brain areas, including the AP, the NTS and parts of the hypothalamus [50, 63, 74, 78, 79, 80].

Recent studies extended our knowledge to non‐human primates and there seems to be a rather large congruence and high degree of conservation across species [75]. Fortunately, we also start to understand receptor distribution in the human brain, in particular, in the hypothalamus and brainstem [81, 82, 83]. The CALCR does not only seem to be expressed in neurons but also in microglia [81]. Surprisingly, in the human brain, more CALCR‐positive neurons seemed to co‐localise with RAMP1 rather than RAMP3 [83], but the latter study did not include analyses of the amount of expression of RAMP1 versus RAMP3 in the respective neurons.

In contrast to the brain, the distribution of functional AMYR is less well characterised in peripheral tissues, in particular when it comes to co‐expression of CTR and RAMPs at the single cell level. In fact, there seems to be limited co‐expression of CALCR and RAMPs in the periphery [84]. CALCR expression has, for example, been reported in the distal convoluted tubule epithelial cells and the thick ascending limb epithelial cells in the kidney, in myeloid cells in the heart and in stem cells of skeletal muscle (satellite cells); RAMP expression is not detected in the latter cells indicating CTR rather than AMYR mediated effects (see also https://shiny.debocklab.hest.ethz.ch/Turiel‐et‐al/). The functional consequence of CTR signalling in satellite cells is currently unknown but may relate to the maintenance of quiescence, muscle regeneration and function [85, 86, 87]. The databases mentioned above [84] provide little information on the bone expression of the AMYR components, but CTR expression in the osteoclasts seems to play an important role in anti‐resorptive effects of amylin [88]; interestingly, these cells seem to specifically also express the AMY2R, but the functional relevance of this specific receptor subtype remains unclear [89]. There is no solid evidence of CALCR and RAMP co‐expression in osteoblasts.

## 4 Systems Integration: Amylin as a Multi‐Organ Network Signal

### 4.1 Islet‐Brain Axis

Pancreatic amylin, together with insulin, acts as a systems‐integrating hormone within the islet‐brain signalling axis. All experimental evidence indicates that amylin's most prominent metabolic effects (induction of satiation; slowing of gastric emptying; inhibition of postprandial glucagon release; increase in energy expenditure; influence on the reward system) are directly brain mediated and do not require signalling via peripheral afferent nerves [4, 29, 37, 90, 91, 92, 93, 94, 95, 96, 97, 98, 99, 100]. Detailed reviews of the central actions of amylin are extensive and beyond the scope of the current review [2, 4, 61, 72, 101]. The following section will only briefly describe some of the key discoveries and conclusions.

Amylin decreases food intake by inducing satiation and, when given chronically, lowers body weight in rodents [27, 98, 102, 103, 104, 105]. These effects seem to depend on functional AMY1R and AMY3R because mice lacking the RAMP1/3 components of the AMYR do not respond to amylin or long‐acting amylin analogs [64, 71, 106, 107]. In addition to reducing energy intake, amylin and amylin analogs also act on the second major component controlling whole body energy balance; in other words, amylin may enhance energy expenditure by counteracting compensatory metabolic adaptations that typically reduce energy expenditure following weight loss; in rats, this effect seems to account for approx. 25% of the reduction of body weight when treated chronically with the amylin analog cagrilintide [105, 108]. The increase in energy expenditure in rodents seems to be at least in part due to increased activation of the sympathetic nervous system to brown adipose tissue [65, 66]. However, given the limited role of brown adipose tissue activity for energy balance in people [109], this mechanism is unlikely to fully translate to humans. Another potential mechanism is by increasing mitochondrial respiration in skeletal muscle [110] and improved muscle contractility in rats by stimulating the Na + pump [111]. Importantly, as mentioned, functional CTR does not seem to be expressed in mature skeletal muscle cells but on stem satellite cells only (see also https://shiny.debocklab.hest.ethz.ch/Turiel‐et‐al/). However, these cells do play important roles in muscle growth and muscle repair, possibly via activation of the sympathetic nervous system [65, 112].

### 4.2 Role of the Caudal Hindbrain in Eating

Components of functional AMYR can be found throughout the brain but we know rather little about amylin effects mediated by many of these receptor sites other than in the hypothalamus and the caudal hindbrain. In fact, co‐expression studies to detect CTR and RAMPs in the same neurons have so far been largely restricted to the AP and hypothalamus [2, 50, 75, 79]. The AP is located in the dorsal vagal complex of the brainstem and is necessary for the acute anorectic effects of amylin as well as body weight regulation [37, 50, 99, 113, 114, 115, 116, 117]. Most systemic effects induced by peripheral amylin administration, such as gastric emptying, food intake, regulation of body weight and potentially also glucagon release, are largely abolished in AP‐lesioned rats. The AP and the neighbouring NTS harbour the necessary AMYR components and have been shown to be directly involved in these effects (see also [75, 118]). AP and NTS neurons project to the lateral parabrachial nucleus (LPBN) and many of these projects seem to be noradrenergic [37, 115, 119]. At least in respect to the involved brain regions, these pathways substantially overlap with those activated by other satiation hormones like glucagon‐like peptide‐1 (GLP‐1) and cholecystokinin (CCK). AP and possibly some NTS neurons express both AMYR and GLP‐1 receptors [75] and therefore can directly activate the same downstream pathway. In contrast, CCK's effect seems to be indirect, via vagal afferents and the NTS, where CCK's action is synergistically modulated by amylin [120, 121, 122].

The LPBN is a crucial relay in the control of feeding but also contains neuronal populations—in particular those expressing the neurotransmitter calcitonin gene‐related peptide (CGRP) that contribute to behaviours associated with visceral illness [118, 123, 124]. CGRP‐positive neurons are not required for amylin‐induced reductions in eating but they are activated by less selective stimuli like sCT [125, 126, 127] which is consistent with the absence of visceral illness by amylin, but not sCT. Interestingly, GLP‐1 also activates CGRP neurons in the LPBN which is probably linked to the stronger negative side effects seen with GLP‐1 receptor stimulation versus AMYR stimulation [75, 118, 128, 129, 130].

### 4.3 Amylin's Role in Hedonic Eating

Beyond homeostatic regulation, amylin also influences hedonic aspects of feeding by modulating reward pathways. Amylin and its analogs seem to affect the rewarding effect of food items [131, 132, 133, 134, 135]. For example, amylin reduces the preference for high fat diet intake in animal models [136, 137] and the amylin analog pramlintide reduces binge eating episodes in humans [138]. Mechanistically, amylin and its analogs seem to reduce dopamine release from neurons in the ventral tegmental area (VTA), potentially by a secondary effect on AP neurons [100]. Various components of the reward system seem to be involved, for example, the VTA—nucleus accumbens (NAc) axis [131, 139, 140], the lateral dorsal tegmental nucleus (LDT) and the medial prefrontal cortex (mPFC) [27, 134, 141]. While the exact circuitry responsible for amylin's effects is not yet defined, it is clear that amylin may not only reduce calorie intake by acting on the homeostatic pathways but also by reducing the rewarding effect of the ingestion in particular of calorie rich food items. The effect may be more generalised and extend to addiction in a broader context because amylin and its analogs also reduce alcohol and nicotine intake in rats [142, 143, 144].

### 4.4 Islet–Brain–Islet Axis

One of the first well established physiological effects of amylin was its modulatory role on pancreatic alpha‐cells by suppressing postprandial glucagon secretion [30, 31, 32]. This effect is most evident under pathophysiological states of Type 1 diabetes mellitus (T1D) and severe T2D, that is, when endogenous amylin is low [9, 145]; in this situation, amylin analogs effectively prevent postprandial hypersecretion of glucagon and hence smoothen postprandial glucose excursions to improve overall glucose control [23, 138].

Seminal experiments showed that amylin infusions exhibited a concentration‐dependent inhibition of the l‐arginine‐stimulated glucagon secretion [32], while infusion of an amylin antagonist induced the opposite effect [31]. It then became clear that the effect of amylin appeared to be indirect because isolated alpha‐cells were not affected by amylin. A central mechanism involving AP neurons is likely but this has not yet been demonstrated unequivocally [146, 147]. In contrast to the marked effects of amylin on glucagon secretion in rats, the effect seems to be less clear in mice. In one study in DIO mice, for example, amylin/leptin co‐administration improved insulin sensitivity but had no clear effect on circulating glucagon levels [148].

Studies in cats have provided additional evidence for the potential therapeutic actions of amylin in regulating pancreatic glucagon secretion in vivo [149, 150]. This is of particular interest because diabetic cats and T2D patients share many pathophysiological similarities so that studies in diabetic cats have a high translational value [17, 151, 152, 153]. In these studies, acute amylin administration reduced plasma glucagon concentrations following intravenous arginine stimulation and mixed‐meal tests. Interestingly, amylin also reduced circulating insulin levels, likely reflecting a lower islet secretory requirement to maintain glycemic control when glucagon output is impeded [149].

The clinical relevance of amylin's beneficial clinical effect on postprandial glycaemia has mainly been studied in humans suffering from T1D and T2D [23, 33, 145, 154, 155]. Using various test paradigms, such as mixed‐meal and standardised test‐meal tests, the amylin analog pramlintide attenuated the postprandial rise in plasma glucagon, which was paralleled with a marked smoothening of glucose excursions compared with the group receiving insulin alone [154, 155, 156, 157]. Hence, pramlintide flattened and delayed the postprandial glucose and glucagon curves and allowed for lower mealtime insulin doses with improved overall glycaemic control. Importantly, amylin's suppression of postprandial glucagon release seems to be equipped with a fail‐safe mechanism because pramlintide's effect on glucagon responses is lost during hypoglycaemia [158], just the same as amylin's effect to slow gastric emptying [30] and its effect to reduce eating [159] in rats. Mechanistically, this fail‐safe mechanism may be due to the co‐sensitivity of AP neurons to amylin and glucose [160]; in other words, ‘sufficient’ glucose levels may be necessary for amylin to act on AP neurons, but this has not been tested formally.

## 5 Combination Pharmacology and Network Effects

Amylin has been shown to functionally interact with several hormones known to affect eating, including CCK, GLP‐1 and estradiol [161]. Among these, only the interaction with CCK seems to be truly synergistic for its short‐term satiating effect [121, 122, 162, 163]. Amylin, GLP‐1 and their analogs have also been shown in numerous studies to reduce eating and eventually body weight more effectively than either compound alone [164, 165, 166]. This combination of treatments has a very high clinical potential, as supported by a large number of recent clinical trials [108, 110, 167, 168, 169, 170, 171, 172, 173, 174, 175].

Amylin and its analogs reduce eating and body weight in males and females; in females, the effect seems to be modulated by estradiol. Interestingly, the influence of estradiol on amylin action differs across the time axis because acutely, estradiol seems to enhance amylin's effect [176] while chronic amylin seems to be more efficacious in animals lacking estradiol, such as shown in ovariectomised female rats [60]. The reason for this differential effect of estradiol is unclear. The clinical implications for anti‐obesity treatment in particular of the latter finding in relation to the menopausal status are currently under investigation.

The most extensive literature for an interaction of amylin with other metabolic hormones exists for leptin. Both hormones are central regulators of energy homeostasis and body weight control [177]. A substantial series of experiments suggests that amylin and leptin act synergistically. The interaction seems to be bidirectional because amylin sensitises the caudal hindbrain and the hypothalamus to the catabolic actions of leptin and the effect of amylin per se also seems to be more prominent in the presence of leptin because amylin's effect is, for example, weakened in leptin‐deficient or leptin‐receptor deficient mice [44, 52, 178, 179, 180, 181, 182, 183, 184].

Mechanistically, amylin may directly enhance leptin receptor signalling and responsiveness in the brain by increasing the expression of leptin receptors in the hypothalamus [44, 50, 178]. Amylin's effect seems to be at least in part mediated via interleukin‐6, which increases leptin‐induced receptor activation and leptin receptor trafficking into the cell membrane in hypothalamic neurons [44, 45, 185].

The translational relevance of this interaction is supported by clinical data. Co‐administration of the amylin analog pramlintide and leptin analog metreleptin in humans resulted in greater weight loss than either treatment alone [186]. In humans, the plasma concentration of the soluble leptin receptor is often used as a proxy to estimate leptin sensitivity in humans [187, 188, 189]. In accordance with the findings reported above, an increase in the concentration of the soluble leptin receptor was observed in a recent clinical trial in people with T2D and treated with cagrilintide but not with semaglutide [169]. Future clinical trials should investigate whether the use of long‐acting amylin analogs sensitises patients to the action of endogenous leptin and whether this effect may contribute to a better maintenance of reduced body weight once weight reduction has been achieved.

## 6 Clinical Translation Beyond Weight Loss—Potential Benefits, Challenges and Open Questions

**FIGURE 4:**
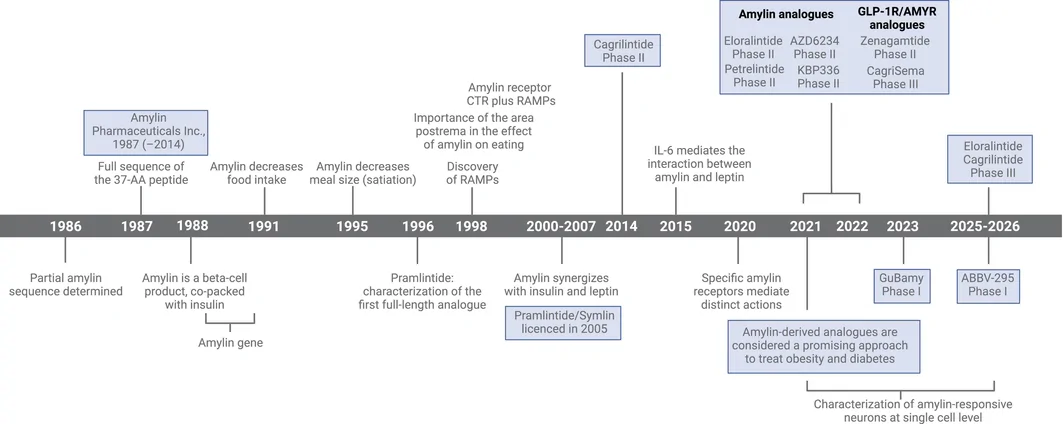
Milestones in amylin research. Timeline of important discoveries in amylin biology, amylin receptor function and the development of amylin analogs. Please note that the dates for clinical trials indicate the time of registration of the trial.

We would like to conclude with a few additional thoughts regarding the role of amylin in the evolving therapeutic landscape against obesity and other metabolic diseases. The timeline for important discoveries in the amylin field is depicted in Figure 4.

Amylin analogs have been claimed to have the potential for a better preservation of lean mass [130, 190] and in particular skeletal muscle mass, compared to GLP‐1 based therapies; preservation of lean mass has come into the focus for anti‐obesity therapy in general [191]. In our understanding, it is probably too early to judge whether this effect and the difference between amylin‐based and GLP‐1 based therapy that has been seen in some pre‐clinical studies, is clinically meaningful. Upcoming studies should definitively address this question. In fact, the most advanced amylin‐based clinical trials looked into the combination of the amylin analog cagrilintide with the GLP‐1 analog semaglutide with very promising efficacy and generally a side‐effect profile that does not seem to extend above that induced by GLP‐1 analogs alone [168, 170, 172, 173]. The pipeline for future amylin‐based therapies is full and will also look into combinations with glucose‐dependent insulinotropic peptide and, for example, with agents directly affecting skeletal muscle biology [6].

Clinical trials with amylin analogs should also investigate in more detail whether improved cardiometabolic function is primarily dependent on the induced weight loss or whether specific effects may offer additional benefit. For example, amylin [192] and its analogs reduce heart rate, in contrast to the increases observed with GLP‐1 analogs [170, 171, 172, 193].

Modern pharmacotherapy has revolutionised the treatment of obesity, with weight reductions exceeding 20% now achievable and likely to improve further with next‐generation drugs [170, 194, 195, 196, 197]. Nonetheless, the major challenge of weight maintenance remains; as has been discussed extensively above [45, 198, 199], leptin sensitisation by amylin analogs may provide additional benefit in sustaining weight loss over time.

Future studies should also address whether specific patient populations may be better treated by amylin analogs than other therapy options. Given that amylin seems to improve memory function [59, 60, 200], it may be speculated that this effect may offer benefits in an elderly population of patients. This population, in particular women, may also benefit from the effects of amylin on bone metabolism and amylin's bone preserving effect (see [2] for review).

Like any other form of pharmacotherapy, amylin‐based drugs do not come without side effects. The most frequent side effects are of gastrointestinal nature but they seem to be milder than with GLP‐1 based medicines [168, 170, 172]. There are also differences among different amylin analogs [130, 201, 202] but the exact reason for such differences—selectivity for AMYR versus CTR; specific characteristics of the receptor‐ligand interactions—still needs to be determined [63, 68, 70].

Other side effects which have been reported in clinical studies may also require our attention in upcoming trials. Fatigue has been reported repeatedly [168, 170, 172, 173], but this may perhaps be a more generalised phenomenon paralleling treatments that lead to substantial weight loss, such as recently also observed with the triple agonist retatrutide which acts on GLP‐1 receptors and receptors for glucose‐dependent insulinotropic peptide and glucagon [197]. In other words, it may not necessarily be a phenomenon linked to the class of AMYR ligands. Further, migraine has been reported after pramlintide administration [203, 204] but it remains unclear if this effect could be due to an interaction with CGRP receptors [205, 206] (and hence may be dose dependent) rather than an amylin‐specific effect.

Finally, we want to emphasise that we only start to understand how well rodent models translate to human amylin biology (amyloid formation, receptor distribution, behavioural assays). It is clear that the basic metabolic phenomena, for example, reduction in eating, lowering of body weight, predictable gastrointestinal side effects, translate very well from rodents (in particular rats) to humans [2]. However, when it comes to more specific questions, such as the affinity of amylin analogs for specific AMYR subtypes and the receptor subtype distribution, more studies are needed to further our understanding. The comparisons of the mouse and the human hypothalamus [82, 207] and of the caudal hindbrain across species [75, 83], are highly interesting and will help design future amylin‐based drugs.

## 7 Conclusion

**FIGURE 5:**
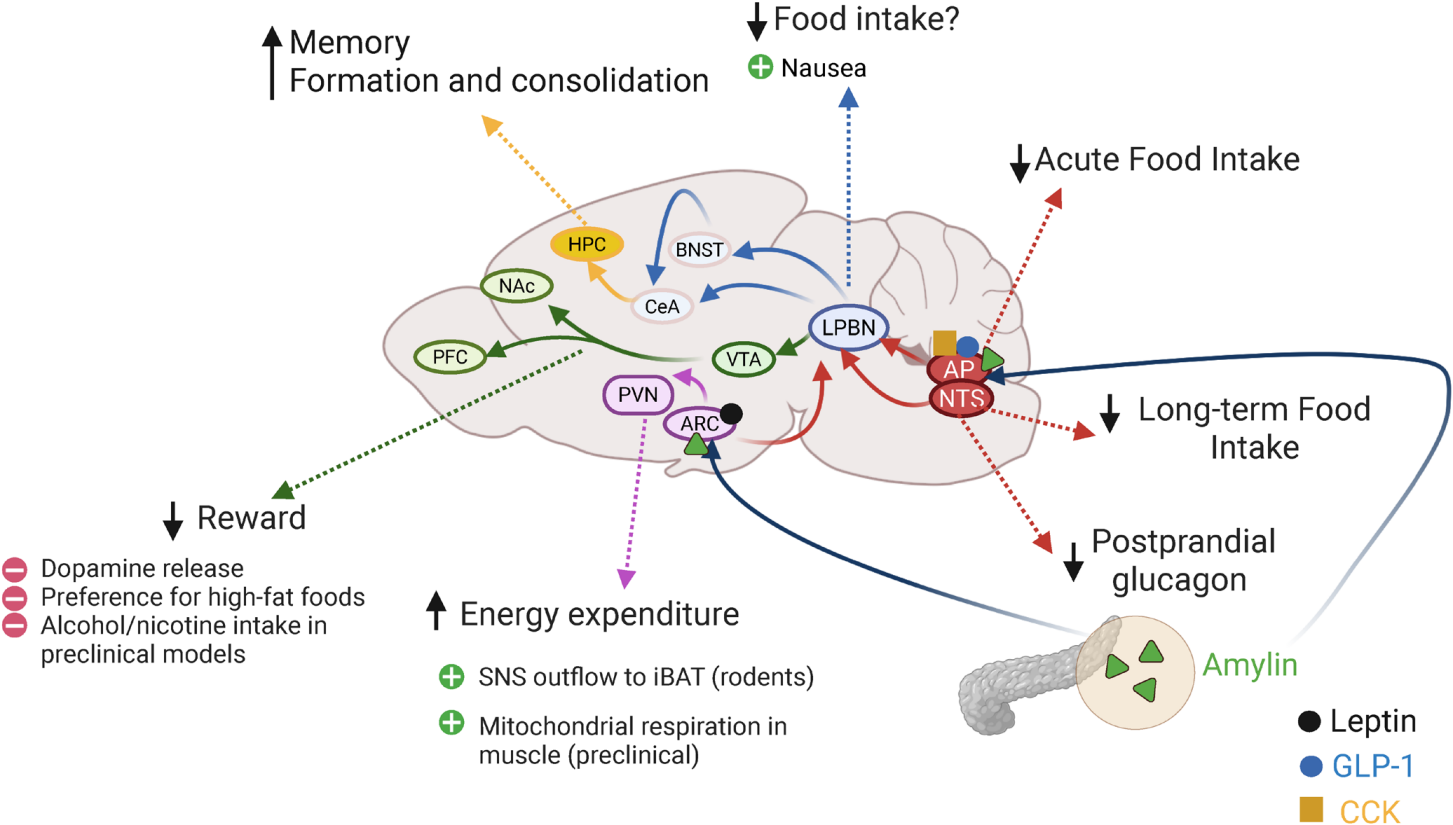
Systems integration and network pharmacology of amylin. Peripheral amylin acts primarily via the AP and adjacent NTS, engaging downstream projections to the lateral parabrachial nucleus, hypothalamus and reward‐related regions to reduce food intake, slow gastric emptying, limit postprandial glucagon secretion and increase energy expenditure. The review further highlights that amylin interacts functionally with CCK, GLP‐1, leptin and estradiol, which reinforce the rationale for combining amylin analogs with GLP‐1 receptor agonists or leptin analogs in clinical strategies for obesity and diabetes. This Figure is an extended version of [61, 208].

In conclusion, pancreatic amylin sits at a systems hub connecting key aspects of energy metabolism (Figure 5). Amylin has an interesting dichotomous function in physiology and pathophysiology. As a circulating hormone, amylin controls satiation, reduces gastric emptying and limits postprandial glucagon release, but its effect on energy metabolism also links homeostatic eating controls with reward‐driven behaviours. On the other hand, aggregating amylin (IAPP) in pancreatic beta‐cells in primates, including humans, and cats has a strong pathophysiological impact on the destruction of pancreatic beta‐cells in T2D and gives a plausible explanation for why a T2D‐like disease entity is only observed in few mammalian species. Our current understanding of the biology of amylin has now been translated into effective pharmacotherapeutic approaches against metabolic diseases.

## Disclosure

Figures were created in BioRender. Le Foll (2026). https://BioRender.com/wv7ndg4.

## Conflicts of Interest

C.L.F. and T.A.L. have a research collaboration with Novo Nordisk. T.A.L. is a consultant with Abbvie, Alveus, AstraZeneca, Curie Bio, Eli Lilly, Gubra, Melogy, Merck, Pila, ProLynx, Protagonist Tx, Roche, Structure Tx, Verdiva, Zealand Pharma.
